# Are individual or group interventions more effective for long‐term weight loss in adults with obesity? A systematic review

**DOI:** 10.1111/cob.12539

**Published:** 2022-06-28

**Authors:** Sarah Street, Alison Avenell

**Affiliations:** ^1^ Health Services Research Unit, Institute of Applied Health Sciences University of Aberdeen Aberdeen Scotland

**Keywords:** adult, group, individual, obesity, systematic review, weight management

## Abstract

Guidelines recommend individual and group interventions for weight loss, based on preference. Our 2009 systematic review compared long‐term effectiveness of individual or group approaches to the same intervention, but there are new randomized controlled trials (RCTs) of high‐quality data. We updated and expanded our previous systematic review. We searched Medline and Embase from 1966 to May 2021, and a clinical trial register from 1966 to 2017. Review Manager (5.4.1) was used to conduct meta‐analysis. Ten RCTs were included. The primary outcome, mean weight change at final follow‐up, was −1.33 kg (95% confidence interval CI: −2.04, −0.62; 10 trials, 2169 participants), favouring group interventions (*p* < .001). For the secondary outcomes, attainment of ≥5% body weight loss at final follow‐up, the risk ratio (RR) was 1.36 (95% CI 1.05, 1.77; three trials, 1520 participants), favouring group interventions (*p* = .02); attrition at final follow‐up was similar between group and individual arms of trials, RR 0.93 (95% CI 0.82, 1.07) (*p* = .31). Group interventions can be more effective than individual interventions for long‐term weight loss in adults with obesity. However, few studies were included in the clinically relevant, secondary outcome measures. Research on delivering group processes in weight management is needed.

## 1 BACKGROUND

Obesity globally is associated with reduced life expectancy,^1^, ^2^ substantial economic impact,^3^, ^4^ and many chronic diseases including heart disease, cancer, and Type 2 diabetes.^1^, ^5^, ^6^ In 2016, 27.8% of UK adults had^7^ obesity and, in 2017, the overall societal cost of UK obesity was estimated at £27 billion annually.^8^

Reducing the prevalence of obesity is a priority^8^ and effective obesity management is therefore essential. Multifaceted lifestyle interventions are known to be effective for weight loss^9^, ^10^ and are recommended by the National Institute for Health and Care Excellence (NICE) in the UK.^11^ A recent systematic review found group interventions including diet and exercise to be effective for long‐term, clinically relevant weight loss.^12^ Current NICE guidelines recommend providing both individual and group interventions and referring individuals to their preferred option.^11^ The guidelines also recommend group over individual interventions where possible, to maximize cost‐effectiveness.^11^

A 2020 systematic review compared individual and group multicomponent weight loss interventions and found group interventions to be significantly more effective for both long‐term weight loss and the attainment of weight loss ≥5% body weight at 12 months.^13^ However, this review included studies that utilized different interventions for individuals and groups in the same study and the comparability of such interventions could be questioned. Our 2009 review compared equivalent individual and group interventions and concluded that group interventions were significantly more effective.^14^ Since then, there have been several large randomized controlled trials (RCTs) of high‐quality data in recent years. The aim of this review is to assess whether individual or group interventions, delivering the same intervention, are more effective for long‐term weight loss for adults with obesity. The aim will be met using the following objectives.

1. A systematic review of the literature across Medline and Embase databases in conjunction with a clinical trial register search and assessment of all studies used in previous systematic reviews, to assess relevant studies from 1966 to the present.
2. Meta‐analysis of weight change and attainment of 5% weight loss to provide revised insight into the effectiveness of the individual versus group comparison for the same weight‐loss interventions for adults with obesity.

## 2 METHODS

### 2.1 Study Selection

A prespecified protocol was followed, registered on PROSPERO (#258665) (https://www.crd.york.ac.uk/prospero/display_record.php?RecordID=258665). Eligible studies were RCTs conducted in adults (mean baseline age ≥18 years) with obesity (mean baseline BMI ≥30 kg/m^2^, or ≥25 kg/m^2^ in ethnic groups with lower BMI cut‐off points),^15^ with a follow‐up period of at least 12 months, including at least one intervention delivered to individuals and groups, with the primary outcome measure being weight change. Interventions had to include a dietary component and be comparable in content for individuals and groups. Exclusion criteria included weight loss medication or surgery, and exercise‐only interventions. Studies published in English‐ and non‐English‐language journals were included, and bibliographic database screening of titles and abstracts was performed independently by both authors.

### 2.2 Literature searching

A literature search of the Medline and Embase databases was conducted from 1966 to 11th May 2021. Search strategies were constructed using a combination of terms for randomized controlled trials, obesity or weight management, and group or individual terminology, and can be found in the Supporting Information Material S1. A clinical trial register^16^ from 1966 to 2017 was also searched for relevant studies, and all studies used in previous relevant systematic reviews were examined and included when relevant.

### 2.3 Data extraction and quality assessment

Data extraction tables were created and completed by one author and all data were checked by the second. Collected data included name of author, publication year, country, setting, intervention details, inclusion/exclusion criteria, qualifications and training of interventionist, session details including total number and time, setting, length of follow up, sample size, gender, ethnicity, mean age and weight/BMI, mean weight change, and the proportion of participants achieving a weight loss ≥5% body weight at follow‐up. Means and standard deviations (SDs) were extracted at baseline and follow‐up for weight (kg) and BMI (kg/m^2^). SDs were calculated from 95% CIs, standard errors, or a prior regression equation when not presented.^17^

If weight change data were not presented, they were calculated from the difference from baseline or BMI change data. All data were entered into Review Manager (5.4.1) by one author and checked by the second before statistical analysis. Quality of evidence was assessed independently by both reviewers using the Cochrane risk of bias 1 tool.^18^ Differences were resolved through discussion between the two reviewers, without recourse to a third reviewer.

The primary outcome was weight change at the final follow‐up. Secondary outcomes were attainment of ≥5% weight loss at final follow‐up, attrition rates at final follow‐up, and cost and cost‐effectiveness data.

### 2.4 Statistical analysis

Review Manager (5.4.1) was used to conduct meta‐analysis to assess the effectiveness of individual versus group interventions, and to calculate heterogeneity represented by the *I* ^2^ statistic.^18^ For both primary and secondary outcome measures, a random‐effects model was used due to expected high heterogeneity (*I* ^2^ > 50%). Data were also analysed at 12 months (eight studies), 18 months (two), 24 months (three), and 36 months (one), and to the last time point of measured weight loss for each study. A continuous inverse variance method was used to calculate the mean difference. A dichotomous Mantel–Haenszel method was used to calculate the risk ratio (RR). Prespecified subgroup analyses were total contact time (higher for group versus equal versus unclear), qualification of interventionist (professional versus non‐professional versus unclear), and in‐person versus remote interventions (in‐person versus remote versus combination). These subgroup analyses were considered important as total contact time could impact the effectiveness and cost‐effectiveness of a study^19^, ^20^; recent studies have shown conflicting results regarding the effect of interventionist qualification on weight loss^21^, ^22^; and remote interventions could be more cost‐effective than in‐person interventions^23^, ^24^ and have become increasingly important, especially in the current pandemic. A blinded outcome assessor (low risk versus unclear/high risk) was included as a sensitivity analysis.

## 3 RESULTS

### 3.1 Study characteristics

**FIGURE 1:**
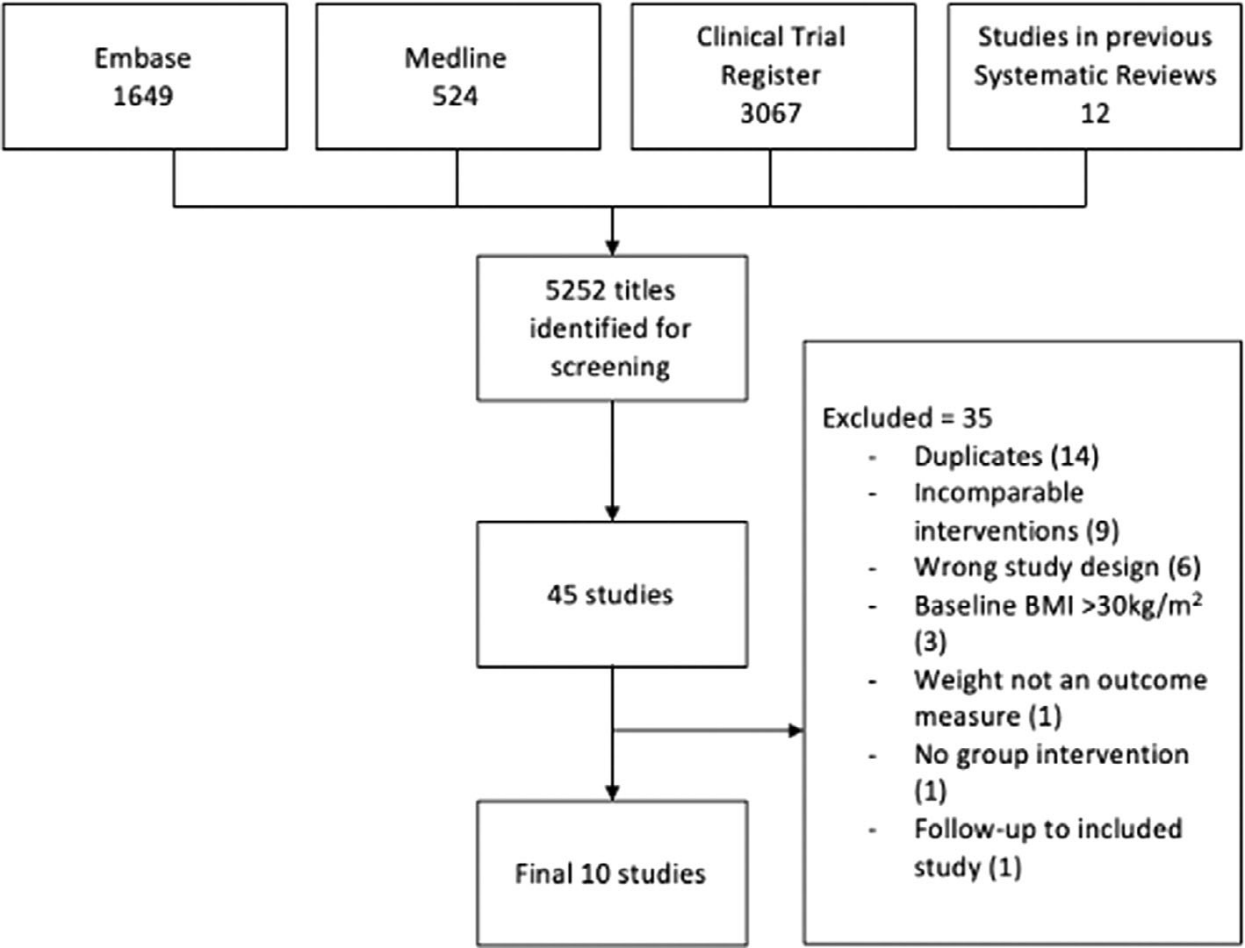
Flow diagram of study selection

The selection process resulted in 10 studies^25^, ^26^, ^27^, ^28^, ^29^, ^30^, ^31^, ^32^, ^33^, ^34^ being included in the systematic review (Figure 1), published between 1977 and 2021. Eight studies were conducted in the USA,^25^, ^26^, ^27^, ^29^, ^31^, ^32^, ^33^, ^34^ one in the UK,^28^ and one in Thailand.^30^ All interventions across nine^25^, ^26^, ^27^, ^29^, ^30^, ^31^, ^32^, ^33^, ^34^ of the 10 studies incorporated calorie reduction, nutritional education, and exercise advice (excluding Jones which did not describe providing exercise advice^28^). Three trials^31^, ^33^, ^34^ described basing their interventions on the Diabetes Prevention Program or Look AHEAD trials. Older trials tended to provide less detail on the behaviour therapy training provided, but all trials described teaching self‐monitoring, all but three trials^27^, ^28^, ^29^ described problem solving, and all but four trials^25^, ^26^, ^27^, ^29^ described goal setting. Five trials described teaching stimulus control^25^, ^26^, ^27^, ^30^, ^33^ and four described cognitive restructuring.^27^, ^29^, ^30^, ^33^ Six of the trials described self‐monitoring and goal setting of physical activity using paper logs, pedometers or other technology.^26^, ^27^, ^31^, ^32^, ^33^, ^34^ Walking was the main form of exercise discussed. None of the trials mentioned physical activity performed in groups.

Studies reported a mean age at baseline ranging from 38.4^30^ to 55.4 years.^32^ Mean BMI at baseline ranged from 28.9^30^ (Southeast Asian) to 39.3 kg/m^2^.^26^ Settings included medical practices^28^, ^29^, ^31^, ^32^, ^34^ (five), community centres^30^, ^33^ (two), and a university^26^ (one), with two papers^25^, ^27^ not reporting on setting. Studies varied from 100% women^25^, ^26^, ^28^, ^29^, ^30^ (five) to mostly (>70%) women^31^, ^33^, ^34^ (three), mostly (>80%) men^32^ (one), and 100% men^27^ (one). Sample size ranged from 26^29^ to 1407.^34^ Length of follow‐up included 12 months^25^, ^26^, ^28^, ^29^, ^30^, ^32^ (six), 18 months^33^ (one), 24 months^27^, ^34^ (two), and 36 months^31^ (one). See Table 1 for baseline characteristics.

**TABLE 1:** Baseline characteristics of included studies

Reference	Study Characteristics	Participants	Interventions
Kingsley and Wilson^25^	Country: USA Setting: NA* Inclusion criteria: Women aged 20–60 years Exclusion criteria: Not being at least 6.8 kg and 10% overweight; involvement in another weight control programme; ongoing psychotherapy; obesity‐related diseases such as diabetes, thyroid dysfunction, colitis, or ulcers; medication that may affect water retention, appetite, or metabolism; pregnancy; unwillingness to commit to the programme or place a deposit. Length of follow‐up: 12 months	Baseline participants (*n*): Total = 78 Individual arm = 26 Group arm = 26 Control = 26 Sex = 100% female Mean age (SD) at baseline, years: Total = 41.5 (NA*) Mean weight (SD) at baseline, kg: Individual arm = NA* Group arm = NA*	Arms: Individual behavioural (a, b) minute group behavioural (a, b) vs. control Interventions details: All participants received the same information on obesity and nutrition, focusing on calorie restriction (1200 kcal/day), increased expenditure and self‐control to create a negative energy balance. Treatment sessions focused on progress review, problem‐solving and self‐control techniques. Participants paid a $55 (USD) deposit and refunds were contingent on attendance, not weight loss. After treatment, 50% of each arm was assigned to four additional booster sessions over the following 14 weeks, with other participants attending only follow‐up weigh‐ins. a. With booster b. Without booster Health professional: Clinical psychology graduate students Total contact time: Individual arm = 8–12 h Group arm = 8–12 h
Straw and Terre^26^	Country: USA Setting: University Inclusion criteria: Women with obesity Exclusion criteria: Body fat percentage <35%, showing signs of serious physical or emotional problems, schedules incompatible with treatment requirements. Length of follow‐up: 12 months	Baseline participants (*n*): Total = 49 Individual standardized arm = 14 Group standardized arm = 14 Control = 14 Sex = 100% female Mean age (SD) at baseline, years: Total = 39.3 (NA*) Mean weight (SD) at baseline, kg: Total = 85.8 (14.73) Individual standardized = 86.7 (16.52) Group standardized = 85.2 (13.97)	Arms: Individual standardized vs. group standardized vs. control Interventions details: All standardized participants received the same behavioural programme in a book with compulsory assignments. Topics included self‐monitoring, stimulus control, eating style, problem solving, activity management and social support. All participants kept food diaries and pedometer records. Health professional: Clinical psychology graduate students Total contact time: Individual arm = 10 h Group arm = 10 h
Jeffery et al.^27^	Country: USA Setting: NA* Inclusion criteria: Self‐reported weight >13.6 kg above ideal weight, self‐report of <6 alcoholic drinks per day Exclusion criteria: Uncontrollable diabetes, heart disease, concurrent dietary or psychological treatment. Length of follow‐up: 24 months	Baseline participants (*n*): Total = 89 Individual arm = 45 Group arm = 44 Sex = 100% male Mean age (SD) at baseline, years: a. Individual $30 = 52.0 b. Individual $150 = 53.8 c. Individual $300 = 52.4 d. Group $30 = 54.1 e. Group $150 = 50.5 f. Group $300 = 53.8 Mean weight (SD) at baseline, kg: a. Individual $30 = 93.1 b. Individual $150 = 99.4 c. Individual $300 = 104.8 d. Group $30 = 96.1 e. Group $150 = 102.9 f. Group $300 = 107.9	Arms: Individual (a, b, c) vs. group (a, b, c) Interventions details: Participants were assigned to groups based on individual deposit amounts (30, 150, or 300 USD) and type of refund (contingent on individual or group performance). All participants received the same advice regarding behaviour (self‐motivation, crisis management), increased exercise, and calorie restriction. Participants kept daily calorie‐intake and exercise records and were weighed weekly. a. $30 deposit b. $150 deposit c. $300 deposit Health professional: NA* Total contact time: Individual arm = 15 h Group arm = 15 h
Jones et al.^28^	Country: UK Setting: Medical centre Inclusion criteria: Women aged ≥18 years, judged suitable by a dietitian. Exclusion criteria: Men, diabetes, pregnancy. Length of follow‐up: 12 months	Baseline participants (*n*): Total = 160 Sex = 100% female Mean age (SD) at baseline, years: Total = 50.3 (NA) Mean weight (SD) at baseline, kg: Individual arm = NA Group arm = NA	Arms: Individual (a, b, c, d) vs. group (a, b, c, d) Interventions details: Individual and group arms were split into the following groups: a. Dietary advice, behavioural leaflet, and monitoring diary b. Dietary advice and behavioural leaflet c. Dietary advice and monitoring diary d. Dietary advice only Dietary advice was given over 5 sessions and included reduced calorie intake (1000 kcal/day or 1000 kcal/day below energy requirements). Health professional: Dietitian Total contact time: Individual arm = 50 min Group arm = 5 h
Wadden et al.^29^	Country: USA Setting: Primary care practice Inclusion criteria: NA* Exclusion criteria: Major illnesses, essential/primary pulmonary hypertension, glaucoma, Type 1 or 2 diabetes, pregnancy, lactation, antidepressant medications, chronic use of nasal decongestants or medications known to affect weight. Length of follow‐up: 12 months	Baseline participants (*n*): Total = 26 Individual arm = 13 Group arm = 13 Sex = 100% female Mean age (SD) at baseline, years: Individual arm = 46.5 (6.1) Group arm = 47.6 (8.5) Mean weight (SD) at baseline, kg: Individual arm = 98.5 (15.6) Group arm = 96.7 (10.4)	Arms: Individual vs. group Interventions details: Delivered in‐person. All patients instructed to consume ~1200 kcal/day, ≤30% kcal from fat, and to increase physical activity. All patients received the same manual and were expected to complete the same weekly assignments. Health professional: Individual arm = psychiatrist Group arm = nutritionist (MSc/PhD) Total contact time: Individual arm = 3 h Group arm = 40 h
Waleekhachonloet et al.^30^	Country: Thailand Setting: Community centre Inclusion criteria: Women aged 20‐60 years, BMI ≥25 kg/m^2^,^a^ an intention to control weight, willingness to follow the protocol, a physically active lifestyle. Exclusion criteria: Medications or products known to affect weight, participation in a weight control programme, uncontrollable diabetes, chronic renal failure, metastasis cancer, dementia, psychiatric diseases, weight loss of ≥5 kg in preceding 6 months, pregnancy, or lactation. Length of follow‐up: 12 months	Baseline participants (*n*): Total = 132 Individual arm = 67 Group arm = 65 Sex = 100% female Mean age (SD) at baseline, years: Individual arm = 38.6 (7.66) Group arm = 38.3 (8.15) Mean weight (SD) at baseline, kg: Individual arm = 70.13 (7.61) Group arm = 69.7 (7.36)	Arms: Individual vs. group Interventions details: All participants were advised to follow a balanced, low‐calorie diet (1200—1500 kcal/day) composed of 15% protein, less than 30% fat, and 55% carbohydrates, in addition to maintaining physical activity habits. All participants received a weight control handbook and dietary information regarding healthy eating, energy balance, portion size, etc. during a meeting at the community centre. Behaviour therapy sessions focused on talking about problems, providing strategies, and reviewing effects. Health professional: Programme providers trained in nutrition, education, and behavioural interventions. Total contact time: Individual arm = 4 h Group arm = 6 h
Weinstock et al.^31^	Country: USA Setting: Primary care practice Inclusion criteria: Age > 18 years, presence of metabolic syndrome based on IDF criteria, BMI ≥30 kg/m^2^ Exclusion criteria: Diagnosed diabetes, presence of severe medical problems that may interfere with participation, e.g., severe ongoing psychiatric illness. Length of follow‐up: 36 months	Baseline participants (*n*): Total = 257 Individual arm = 129 Group arm = 128 Sex: Individual arm = 78.3% female Group arm = 71.9% female Mean age (SD) at baseline, years: Individual arm = 50.7 (13.1) Group arm = 52.7 (12.8) Mean weight (SD) at baseline, kg: Individual arm = 105.8 (23.6) Group arm = 109.4 (26.1)	Arms: Individual remote vs. group remote Interventions details: Diabetes Prevention Program (DPP) materials were delivered by telephone with written materials provided at baseline visits. Educators followed scripts including goal setting, self‐monitoring, diet/activity modification and problem‐solving. For the group intervention, scripts included prompts for educators to engage all group members in the discussion. Health professional: Nurses and medical office assistants. Total contact time: Individual arm = NA* Group arm = NA*
Damschroder et al.^32^	Country: USA Setting: Medical centre Inclusion criteria: Obesity, at least one obesity‐related chronic health condition without contraindications for weight loss, English speaking, competency to provide informed consent, reliable access to a telephone Exclusion criteria: Current involvement in another similar study, ongoing treatment or medication for weight loss, inability to complete the 6‐min walk test, pregnancy. Length of follow‐up: 12 months	Baseline participants (*n*): Total = 481 Individual arm = 162 Group arm = 160 Control = 159 Sex = Individual arm = 84% male Group arm = 83.8% male Control = 87.4% male Mean age (SD) at baseline, years: Individual arm = 55.4 (10.0) Group arm = 54.9 (9.5) Control = (54.6 (10.5) Mean weight (95% CI) at baseline, kg: Individual arm = 112.5 (109.1, 116.0) Group arm = 112.4 (109.0, 115.8) Control = 114.1 (110.4, 117.8)	Arms: Individual vs. group vs. control Interventions details: Participants in individual or group arms received manuals with session content and were encouraged to log daily dietary intake using the Stoplight Guide, which categorizes foods as red (high‐calorie, low nutrition); yellow (high‐calorie, high nutrition); or green (low‐calorie, high nutrition). Advice also included tracking physical activity through daily pedometer use and weighing oneself weekly. Coaching sessions provided progress reviews, problem‐solving and the setting of small, manageable goals. The control group received different informational handouts, pedometers, and food intake logbooks. Health professional: Lifestyle coach with BSc and in‐house training Total contact time: Individual arm = 10–12 h Group arm = 34 h Control = 36 h
Perri et al.^33^	Country: USA Setting: Community centre (Cooperative Extension Service site) Inclusion criteria: Age = 21‐75 years, BMI 30–45 kg/m^2^, free of uncontrollable diabetes and hypertension, no active manifestations of cardiovascular, cerebrovascular, renal, or hepatic disease. Exclusion criteria: Use of medications known to affect body weight, musculoskeletal conditions that preclude walking for 30 min, weight loss >4.5 kg in preceding 6 months, psychological contraindications including depression and substance abuse. Length of follow‐up: 18 months	Baseline participants (*n*): Total = 445 Individual arm = 149 Group arm = 143 Control = 153 Sex = 82.7% female Mean age (SD) at baseline, years: Individual arm = 55.9 (10.2) Group arm = 55.4 (9.8) Control = 54.8 (10.7) Mean weight (95% CI) at baseline, kg: Individual arm = 90.4 (87.9, 92.9) Group arm = 93.3 (90.7, 95.9) Control = 90.6 (88.6, 92.7)	Arms: Individual vs. group vs. control Interventions details: Content addressed challenges commonly experienced in rural areas (traditional high‐calorie cooking, a lack of community exercise facilities). Calorie restriction was advised, with caloric goals based on weight. Participants were instructed to keep daily logs of consumed foods and corresponding caloric values and to increase planned daily walking by 3000 steps. For individual and group arms, health coaches offered support, encouragement, and feedback. For control, materials were sent via email or post with no contact. Health professional: In‐house agent or individual with relevant BSc/MSc Total contact time: Individual arm = 3–6 h Group arm = 18 h
Befort et al.^34^	Country: USA Setting: Primary care Practice or remotely Inclusion criteria: Age = 20–75 years, BMI = 30–45 kg/m^2^, reside in rural location, visited a clinic at least once in prior 18 months Exclusion criteria: History of bariatric surgery, pregnancy, myocardial infarction or stroke, new cancer diagnosis in last 6 months. Length of follow‐up: 24 months	Baseline participants (*n*): Total = 1407 Individual arm = 473 Group arm = 468 Control = 466 Sex = 76.8% female Mean age (SD) at baseline, years: Total = 54.7 (11.8) Mean weight (SD) at baseline, kg: Individual arm = 103.1 (15.4) Group arm = 102.9 (15.5) Control = 102.7 (15.6)	Arms: In‐person individual vs. in‐person group vs. control Interventions details: All participants received the same recommendations on diet, physical activity, and behaviour change strategies. Advice included consuming a low‐calorie, balanced diet with 5+ fruit and vegetable servings per day and increasing exercise to 225 min per week. Calorie goals were based on weight and participants were advised to set weekly goals and self‐monitor daily. Health professional: Individual arm = clinician Group arm = clinician Total contact time: Individual arm = 8 h Group arm = 36 h Remote group arm = 36 h

^a^ Southeast Asian population so BMI ≥25 kg/m^2^ is considered obese.

* NA, not currently available.

### 3.2 Risk of bias

The first Cochrane risk of bias tool was used to assess the quality of evidence (Table S1). One study was high risk in four of the seven areas^34^; the other nine studies were of high risk in two or less areas.^25^, ^26^, ^27^, ^28^, ^29^, ^30^, ^31^, ^32^, ^33^ Allocation concealment was of unclear risk for nine studies,^25^, ^26^, ^27^, ^28^, ^29^, ^30^, ^31^, ^32^, ^34^ with one study at high risk.^33^ All studies were at high risk of bias for blinding participants and personnel, which is common for nutritional interventions. Selective reporting was of unclear risk for eight studies,^25^, ^26^, ^27^, ^28^, ^29^, ^30^, ^31^, ^32^ with two studies at low risk.^33^, ^34^ Ignoring high risk of bias for blinding of participants and personnel, no study was otherwise all at low or all at high risk of bias for all other domains. To review small study bias, Review Manager (5.4.1) was used to assess funnel plot symmetry (Figure S2). No gross asymmetry was observed; however, 10 studies are marginally sufficient to perform this assessment.

### 3.3 Outcome results

**FIGURE 2:**
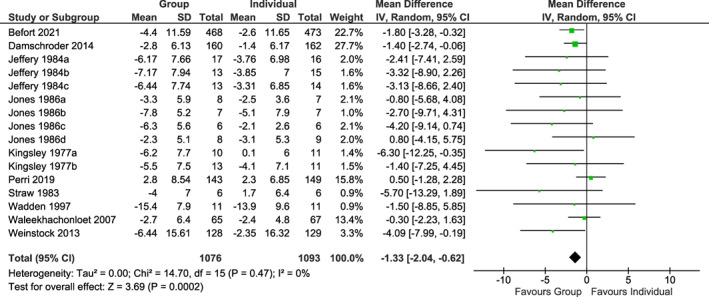
Mean weight change at final follow‐up in kg

The mean weight change was −1.36 kg at 12 months (95% CI −2.27, −0.45; 8 trials,^25^, ^26^, ^27^, ^28^, ^29^, ^30^, ^31^, ^32^ 937 participants); −0.95 kg at 18 months (95% CI −3.69, 1.79; two trials,^33^, ^34^ 1233 participants); −2.26 kg at 24 months (95% CI −3.51; −1.02; 3 trials,^27^, ^31^, ^34^ 1286 participants), and − 4.09 kg at 36 months (95% CI −7.99, −0.19; 1 trial,^27^ 257 participants), all favouring group interventions. The mean weight change at final follow‐up (primary outcome) was −1.33 kg (95% CI −2.04, −0.62; 10 trials,^25^, ^26^, ^27^, ^28^, ^29^, ^30^, ^31^, ^32^, ^33^, ^34^ 2169 participants), favouring group interventions (*p* < .001) (Figure 2). For one study, data were used for all intervention arms from the beginning of the study, as data from randomization were not available.^33^

**FIGURE 3:**
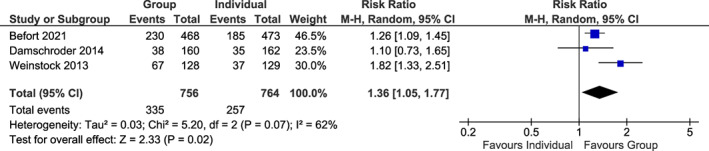
Attainment of ≥5% weight loss at final follow‐up

Only four studies^31^, ^32^, ^33^, ^34^ reported the secondary outcome measure, attainment of weight loss ≥5% body weight at final follow‐up, and only three of these studies were included in the analysis as the fourth study measured this outcome from baseline not from time of randomization to interventions.^33^ For the attainment of weight loss ≥5% body weight at final follow‐up, the RR was 1.36 (95% CI 1.05, 1.77; three trials,^31^, ^32^, ^34^ 1520 participants), favouring group interventions (*p* = .02) (Figure 3). For the number of dropouts at final follow‐up, the RR was 0.93 (95% CI 0.82, 1.07; eight trials,^25^, ^28^, ^29^, ^30^, ^31^, ^32^, ^33^, ^34^ 2182 participants) with similar attrition rates between group and individual arms of trials (*p* = .31) (Figure S1).

Of the 10 studies, four^28^, ^29^, ^31^, ^34^ used interventionists with professional qualifications, three^25^, ^26^, ^32^ used students or non‐professionals, and three^27^, ^30^, ^33^ were unclear. Five studies^25^, ^31^, ^32^, ^33^, ^34^ mentioned in‐house training, which varied from a half‐day to 3 days plus ongoing reviews during the trials. Mean weight change (95% CI) in interventions delivered by a professional was −1.97 kg (−3.18, −0.75; *p* = .001) compared to −2.22 kg (−4.27, −0.16; *p* = .03) for a non‐professional and − 0.33 kg (−1.54, 0.87; *p* = .59) for unclear studies. Subgroup differences were not statistically significant (*χ* ^2^ = 4.41, *df* = 2, *p* = .11).

Four studies blinded outcome assessors,^25^, ^30^, ^31^, ^33^ six studies either did not mention blinding or did not blind outcome assessors.^26^, ^27^, ^28^, ^29^, ^32^, ^34^ Mean weight change (95% CI) in studies that utilized blinding was −1.33 kg (−3.36, 0.70; *p* = .20) compared to −1.74 kg (−2.62, −0.86; p < 0.001) in all other studies. The difference was not statistically significant (*χ* ^2^ = 0.13, *df* = 1, *p* = .71).

Although components in interventions within studies were the same, contact time varied greatly: three studies^25^, ^26^, ^27^ gave equal contact time to individual and group interventions, while total contact time was greater (from ×1.5 to ×13) for group than individual interventions in six studies.^28^, ^29^, ^30^, ^32^, ^33^, ^34^ One study did not report contact time.^31^ Mean weight change (95% CI) in studies with equal contact time was −3.47 kg (−5.83, −1.11; p < 0.01) compared to −1.01 kg (−1.77, −0.25; p < 0.01) for studies with higher contact times for groups than individuals. Subgroup differences were not statistically significant (*χ* ^2^ = 5.77, *df* = 2, *p* = .06).

Seven studies^25^, ^26^, ^27^, ^28^, ^29^, ^30^, ^34^ delivered interventions in‐person, two studies^31^, ^33^ delivered interventions remotely, and one study utilized a combination.^32^ Mean weight change (95% CI) for interventions delivered in‐person was −1.67 kg (−2.64, −0.70; p < 0.001), compared to −1.45 kg (−5.90, 3.00; *p* = .52) for remote interventions and − 1.40 kg (−2.74, −0.06; *p* = .04) for a combination. Subgroup differences were not statistically significant (*χ* ^2^ = 0.11, *df* = 2, *p* = .95).

In a post‐hoc subgroup analysis, mean weight change (95% CI) in two trials^27^, ^32^ with interventions delivered to predominantly male groups was −1.64 kg (−2.87, −0.41; p < 0.01), compared to −1.34 kg (−2.43, −0.24; *p* = .02) for interventions delivered predominantly to women in eight trials.^25^, ^26^, ^28^, ^29^, ^30^, ^31^, ^33^, ^34^ Subgroup differences were not statistically significant (*χ* ^2^ = 0.13, *df* = 1, *p* = .72).

### 3.4 Economic data

Two studies reported on cost and/or cost‐effectiveness data. Waleekhachonloet et al.^30^ reported in 2007 that the group behaviour therapy required 66 h of staff time versus 156 for the individual behaviour therapy, and total programme costs were $524.5 USD for group behaviour therapy versus $767.5 USD for individual behaviour therapy. Conversely, Wadden et al.^29^ reported in 1997 that each 1‐kg reduction in body weight cost $182 USD in the individual arm versus $246 USD in the group arm, including patient time as a cost.

## 4 DISCUSSION

Our results continue to suggest that group interventions can be more effective than individual interventions for long term, clinically relevant weight loss in adults with obesity. However, only three studies were included the secondary outcome measure (attainment of weight loss ≥5% body weight), so care must be taken when interpreting the clinical significance of this result. None of the subgroup analyses had statistically significant effects on long‐term weight loss; the limited number of studies would have impacted these results. Additionally, the five older studies included in this review (published 1977–1997) all omitted important information relating to the risk of bias assessments or information needed for subgroup analyses, as did three of the five newer studies (published 2007–2021). Two of the five subgroup analyses, therefore, had unclear or unknown categories, and this lack of clarity may have also affected overall findings. No difference was demonstrated in attrition rates between interventions at final follow‐up (RR = 0.93, 95% CI 0.82, 1.07, *p* = .31), meaning no attrition bias could be detected. Attrition rates within studies varied from ~9% to ~64% and were highest in older^28^ or in longer^31^ studies, as would be expected.

Our 2009 review^14^ concluded that group interventions were more effective than individual interventions for long‐term weight loss in the target population (−1.4 kg; 95% CI −2.7, −0.1). Confidence intervals were wider than our findings here, and the effect size was slightly larger. Our update includes substantially longer durations of follow‐up than the 2009 review, of which the longest follow‐up time was 12 months.

A 2020 systematic review^13^ also found group interventions to be significantly more effective than individual interventions, both for total weight change (−1.9 kg; 95% CI −2.6, −1.3) and for the attainment of weight loss ≥5% body weight (RR = 1.58; 95% CI 1.25, 2.00). This represents a larger effect size and slightly narrower confidence intervals than our review. However, the 2020 review included studies which utilized different interventions for individuals and groups, asking a different question from our update, not solely individual versus group approaches for the same intervention. There was no overlap in included studies between this review and our updated review.

A systematic review by Borek et al.^12^ from 2018 also evaluated group‐based diet and physical activity weight loss interventions, comparing interventions from 47 RCTs to no interventions, weight list controls, minimal interventions, or usual care. Although the mean difference in weight loss at 24 months was −2.56 kg (95% CI −3.79, −1.33), this difference again does not reflect the difference in intervention delivery, which we examined here. However, these investigators went on to develop the MAGI framework (mechanisms of action in group‐based interventions).^35^ The MAGI framework highlights the pivotal importance of facilitation techniques and contextual influences for behaviour change in groups, emphasizing the importance of selection and training of facilitators in future interventions.

Our results complement the conclusions of these reviews, although our findings suggest a smaller effect size. This may in part be due to the longer follow‐up time of our review, as weight loss maintenance is challenging, and initial weight loss is usually regained over longer periods.^36^ It is also a function of only including comparable interventions, suggesting our results reflect the actual difference between individual and group weight‐loss interventions over time.

Our subgroup analysis agrees with recent findings that interventionist qualifications have no significant effect on long‐term weight loss,^21^ which might indicate that the quality of relevant training is more important. However, a 2017 systematic review found dietitian‐led weight loss interventions to be significantly more effective than non‐dietitian‐led interventions.^22^ Our findings may be due to our inclusion of clinicians, psychologists, and other specialists within the professional qualification category. Future studies could differentiate dietitians from other specialists.

Strengths of our review include the long follow‐up time (12–36 months) and only including comparable interventions. Our review includes studies involving only men, only women, and various percentages of both, increasing the generalizability of results.

Limitations of our review include the fact that nine of the 10 studies were conducted in Western countries, eight from the USA, and results may therefore not be generalizable to other countries or cultures. Whether trials were representative of people with obesity in Western countries is unclear. Only four studies provided information on ethnicity^31^, ^32^, ^33^, ^34^ or markers of socioeconomic status such as income or education^31^, ^32^, ^33^, ^34^; three of these studies^32^, ^33^, ^34^ reported recruiting from disadvantaged communities. The socio‐economic status of participants could be an important factor in the long‐term successfulness of weight‐loss interventions due to environmental and psychological factors such as social resources or stress.^37^ Results for costs and cost‐effectiveness from only two studies were contradictory.^29^, ^30^ However, previous studies have suggested, and current guidelines state, that group interventions are often more cost‐effective than individual interventions.^11^, ^24^, ^30^ It would be helpful to have more data on cost and cost‐effectiveness from future trials.

It was not within the scope of this review to assess group interactions or composition or to explore why group interventions were more effective than individual interventions, yet group dynamics could be key factors in performance and success rate.^38^ With only 10, mostly small trials, we had the very limited statistical power to examine these factors in subgroup analyses. Before recommending group over individual interventions on a national scale, more research is needed on specific commonalities of groups successful in long‐term weight loss. For example, perceived group conflict is associated with reduced weight loss and higher group attraction is associated with higher attendance rates,^39^ an important factor in long‐term weight loss success.^40^, ^41^ Whether it is possible to foster low perceived group conflict and high group attraction, and whether this would promote greater weight loss, are interesting questions for future research. Furthermore, a recent systematic review demonstrated that the participant‐interventionist bond was significantly associated with greater weight loss and adherence, while participant expectations and programme type showed no significant association.^42^ This suggests that, rather than focusing on specificities of the intervention itself, a more important focus may be who is conducting the intervention, and the training they receive. While this review attempted to assess the effect of interventionist type on total weight loss, no association was found, potentially due to the small number of studies. A study powered for this objective may be helpful to maximize the effectiveness of future weight loss interventions. Lastly, as it is well‐documented that long‐term reversal of obesity is extremely challenging,^43^, ^44^ it may be valuable to conduct a similar review on weight‐loss in adults with overweight rather than obesity, emphasizing prevention rather than reversal.

## 5 CONCLUSION

Group interventions can be more effective than individual interventions for long‐term weight loss in adults with obesity. However, few studies were included in the clinically relevant, secondary outcome measure of attainment of ≥5% body weight loss at final follow‐up. Research on delivering group processes in weight management is needed.

## CONFLICT OF INTEREST

The authors declare no conflicts of interest.

## Supporting information


**Figure S1** Number of dropouts in group versus individual interventions at final follow‐up.
**Figure S2**. Funnel plot of weight change (kg) at final follow‐up.
**Table S1**. Finalized risk of bias 1 assessments.
