# Supplementary material for: Are individual or group interventions more effective for long‐term weight loss in adults with obesity? A systematic review
Source: Clin Obes. 2022 Jun 28;12(5):e12539. doi: 10.1111/cob.12539 (PMC9542282; doi:10.1111/cob.12539)
Supplement: Supplementary file 1 — Figure S1 Number of dropouts in group versus individual interventions at final follow‐up. Figure S2. Funnel plot of weight change (kg) at final follow‐up. Table S1. Finalized risk of bias 1 assessments. [file COB-12-e12539-s001.docx]

**Appendix**

Figure S1. Number of dropouts in group versus individual interventions at final follow-up.


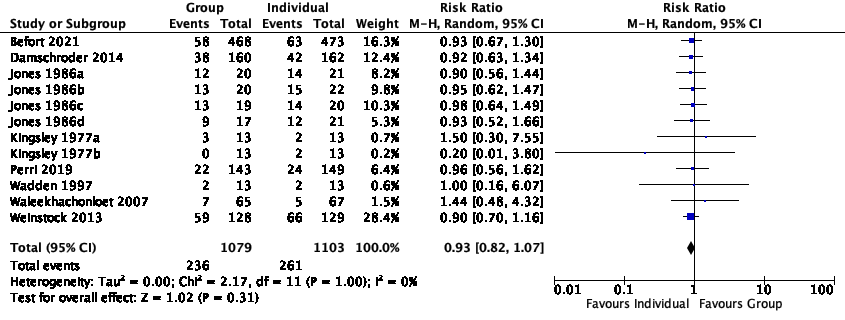


Table S1. Finalised Risk of Bias 1 Assessments.

| Studies | Random Sequence Generation | Allocation Concealment | Blinding Participants & Personnel | Blinding of Outcome Assessment | Incomplete Outcome Data | Selective Reporting | Other Bias |
| --- | --- | --- | --- | --- | --- | --- | --- |
| Kingsley | Unclear | Unclear | High | Low | Low | Unclear | Low |
| Straw | Unclear | Unclear | High | Unclear | High | Unclear | Unclear |
| Jeffery | Unclear | Unclear | High | Unclear | Unclear | Unclear | Unclear |
| Jones | Unclear | Unclear | High | Unclear | High | Unclear | Unclear |
| Wadden | Unclear | Unclear | High | Unclear | Low | Unclear | High |
| Waleekhachonloet | Unclear | Unclear | High | Low | Low | Unclear | Low |
| Weinstock | Unclear | Unclear | High | Low | High | Unclear | Low |
| Damschroder | Low | Unclear | High | High | Unclear | Unclear | Low |
| Perri | Low | High | High | Low | Low | Low | Low |
| Befort | Low | Unclear | High | High | High | Low | High |

Figure S2. Funnel plot of weight change (kg) at final follow-up


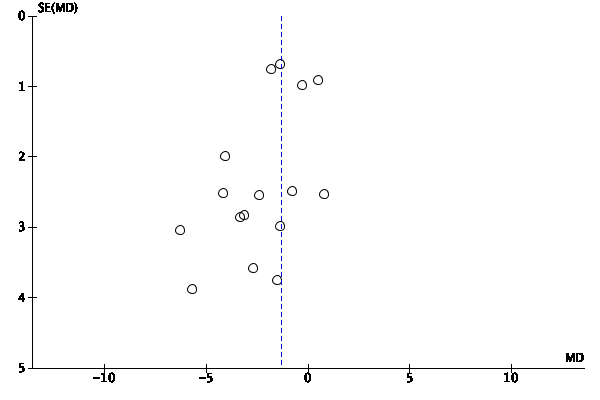


Full search strategy for EMBASE (OVID) database.

| 1     clinical trial/ (883825) 2     Multicenter Study/ (281380) 3     phase 2 clinical trial/ (82572) 4     phase 3 clinical trial/ (52259) 5     phase 4 clinical trial/ (4105) 6     Randomized Controlled Trial/ (618990) 7     controlled study/ (7622243) 8     meta analysis/ (212289) 9     crossover procedure/ (63119) 10     double blind procedure/ (160133) 11     single blind procedure/ (41495) 12     randomization/ (83267) 13     Major Clinical Study/ (3364398) 14     placebo/ (317998) 15     drug comparison/ (2097) 16     clinical study/ (105630) 17     (clin$ adj25 trial$).tw. (673762) 18. ((singl$ or doubl$ or tripl$ or trebl$) adj25 (blind$ or mask$)).tw. (215862) 19     placebo$.tw. (279999) 20     random$.tw. (1544257) 21     control$.tw. (4590614) 22     or/1-21 (12241929) 23     22 and nonhuman/ (3437406) 24     22 not 23 (8804523) |  |
| --- | --- |
| 25. obesity/ |  |
| 26. obesity in diabetes/ or obesity, morbid.mp. |  |
| 27. hyperphagia/ or bulimia/ |  |
| 28. obes$.mp. |  |
| 29. weight loss.mp. |  |
| 30. overweight.tw. |  |
| 31. (weight adj1 (maint$ or reduc$)).tw. |  |
| 32. (los$ adj1 weight).tw. |  |
| 33. (diet$ adj5 weight).tw. |  |
| 34. (weight adj1 control).tw. |  |
| 35. or/25-34 |  |
| 36. limit 35 to (embryo or infant or child or preschool child <1 to 6 years> or school child <7 to 12 years> or adolescent <13 to 17 years>) |  |
| 37. 35 not 36 |  |
| 38. 24 and 37 |  |
| 39. group$.ti. |  |
| 40. individual$.ti. |  |
| 41. 38 and 39 and 40 |  |
